# To Dr. Dickson, Fellow of the Royal College of Physicians. Edinburgh, and Physician to the Fleet

**Published:** 1816-11

**Authors:** John Hall


### *For the London Medical and Physical Journal.*

[We have been favoured with a Copy of the following Letter on the Depletory Treatment of Fever; by JOHN HALL, M.D. of Berwick. And also with some Remarks by Dr. DICKSON.]

*To Dr. Dickson, Fellow of the Royal College of Physicians. Edinburgh, and Physician to the Fleet.*

MY DEAR SIR,

YOUR observations published in the Edinburgh Medical and Surgical Journal of April, on the utility of blood-letting and purgatives in a fever which prevailed in the Russian fleet, gave me the highest satisfaction, not only as they proved the beneficial effects of general depletion in the early stage of fever, but as they tended to confirm, in my own mind, doctrines that had been inculcated in the early part of my life. These, notwithstanding I adopted the practice of the times in which I have lived, I feel pleasure in saying I have never totally lost sight of; and oft has been the time when, in my own opinion, I should have felt justified in deviating from the general practice in the treatment of fevers, that I have been imperiously withheld by an implicit veneration for authorities, which I had been taught to consider as the highest standard of medical practice. That my conduct, however, has not been actuated by this motive, the case which I shall have occasion to mention afterwards, besides many others which I could adduce, will sufficiently prove; and from the successful result of such I have had no cause to repent of my deviation.

From your other valuable papers that have lately been published upon this subject, and the beneficial effects of such practice being so generally admitted, I should not have intruded these observations upon your notice at present, did I not feel it a duty which I owe to the memory of my deceased father, (many years ago a practitioner in this place, and with whom I received the early rudiments of my medical education,) to mention that such were his doctrines of fever, as evinced by his practice, to the success of which, as far as my memory can refer, I can bear testimony. At that time, I could not reason upon or judge of its propriety, but upon going to Edinburgh I became a convert to the doctrine of debility, &c.; and was the more surprised from its generally acknowledged preference to other theories, that any patients should have recovered under the depleting system. A purge of jalap and calomel was the principal remedy which my father used in fever, generally preceded by bleeding. He seldom seldom gave emetics in a full dose, but regularly after the cathartic. He prescribed nauseating doses of tartarized antimony, which seldom failed of operating powerfully both upwards and downwards; and, unless where the fever had run some days previously, of producing a favourable crisis. Opiates he seldom used, or, if he did, it was in such small doses as to be incapable of doing either much good or harm. Cinchona he never administered until a crisis was obtained, or towards the close of the disease, when wine was also allowed.

This, with some little exception, appears to be the present mode of treating fever, and certainly it is much preferable to that powerfully stimulating plan so prevalent some years ago, to which the doctrines of the day so much contributed; and it leads me to conclude with you, that most cases of typhus are originally synochas, and become typhoid, either from being permitted to run on, or from improper treatment in the beginning: indeed, pure typhus is probably almost as rare as pure synocha.

Many of the symptoms which are so strongly marked in the progress of those fevers which terminate fatally, I am inclined to think would have been altogether prevented by early depletion and evacuations, or so modified as to render their future treatment more manageable; whereas, by an opposite practice, congestion, effusion, or disorganization of some important viscus is induced, and the very aids which we offer in the latter stages of fever too often, I fear, precipitate the fatal event.

In one case of fever this took place in consequence of a profuse hæmorrhage from the bowels. It was not preceded by any topical affection whatever, or symptom, but those of general fever, and was treated as typhus. From the event which happened as sudden as unexpected, I would infer, that, if depletion had been used in the early period of the disease, congestion (probably in the liver) which took place, and led to this fatal occurrence, might in all probability have been prevented.

I shall now subjoin the case, above alluded to, in illustration of the different value of these different modes of practice in fever. Believe me, my dear Sir,

Your's sincerely,

(Signed) John Hall.

Case.—About three years ago I was sent for to a house to see Mrs. B. She had been unwell for two days, but, from being subject to head-ach, did not particularly attend to it. When I saw her she complained of a violent pain in her head, accompanied with a sense of tightness, throbbing of

 the the temples, and she could with difficulty distinguish objects. Typhus fever was then prevailing in the village of L——, and apprehensions were expressed that some of the servants might have incautiously visited in some of the houses of those labouring under this disease. Be that as it may, little doubt could be entertained as to the practice proper in the present case; and, accordingly a very full bleeding was used with the greatest and most immediate relief, so much so, that during the operation she told me that she could then see me distinctly, which she could not do before. It was followed up by a purge of jalap and calomel, and subsequently with small doses of calomel and antimonial powder; and in a few days she was convalescent. Upon taking my leave at this time, I specified the day on which I should return, but from my horse becoming lame, and not conceiving it of consequence whether I saw her on that or the next day, I did not make out my visit as I at first intended. In the mean time Miss W. B. was taken ill, but, as I was expected according to my promise, no intimation of it was given to me; and, not making my appearance in the evening, a physician who happened to be in the neighbourhood was sent for to see her. Upon learning that typhus fever was in the village, and that his patient had attended upon her mother, he pronounced it to be that disease, to the great consternation of the family. No doubt now remained that their former suspicions respecting the servants having brought the contagion from the village were well founded; and a system of fumigation and quarantine was adopted. In the mean time my patient was up and going about attending her daughter; whilst the latter, who appeared to have a mild disease, had a protracted recovery, and was nearly a month before she was able to leave her room.

The remarks I would make in the two cases before me are, that I have no doubt of the fever having been the same in both; that it had been produced in each by specific contagion; and that the first case would have terminated in an aggravated form of the disease, and perhaps fatally, if it had not been arrested by powerful depletion, which led to a remission of febrile action, subsequent convalescence, and rapid recovery. Whereas, the latter was treated, probably in consequence of the disease being milder, by purgatives and diaphoretics in the first instance, allowing the fever to run its usual course; and afterwards, by decoct. cinchonæ acid. sulphuric. delut., wine, &c.—thus walking by rule, and conforming strictly to nosological definition.

*Berwick-upon-Tweed;*

17*th of September,* 1816.

