# Corrigenda

**Published:** 1816-12

**Authors:** 


					p. 368, I. 16 of the letter,
tor " has not been actuated," read " has not been always actuated;
j). 368, I. 21 of the letter, for " From your other valuable papers," read
" From your, and several other valuable papers;" p. 369,1.16, for synochas
read synochus; p. 370, last line but one, for delut. read dilut.;

				

